# Thyroid metastasis from small cell lung carcinoma: a case report and review of the literature

**DOI:** 10.1186/s13256-015-0707-4

**Published:** 2015-10-07

**Authors:** Ahmet Selçuk Can, Gülistan Köksal

**Affiliations:** 1 Termal Vocational School, Yalova University, Gökçedere Mahallesi, Kışla Caddesi, Nergis Sokak, No: 23, Termal, Yalova 77200 Turkey; 2 Division of Oncology, Department of Internal Medicine, Private Gayrettepe Florence Nightingale Hospital, Istanbul, Turkey

**Keywords:** Neoplasm metastasis, Small cell lung carcinoma, Thyroid metastasis

## Abstract

**Introduction:**

Small cell lung carcinoma frequently metastasizes to lymph nodes, liver, adrenal glands, bone, brain and pleura. Metastasis of small cell lung cancer to the thyroid gland is extremely rare.

**Case presentation:**

A 55-year-old Turkish man presented with a mediastinal mass intermingled with mediastinal lymphadenopathy, measuring 11cm in total, and encasing superior vena cava and deviating his trachea, esophagus and vascular structures. He had superior vena cava syndrome. His thyroid appeared normal on computed tomography of his chest. A bronchoscopic biopsy showed small cell lung carcinoma. Chemotherapy with cisplatin and etoposide and external radiotherapy was given. Six months after the presentation, multiple brain metastases were detected on magnetic resonance imaging. Chemotherapy was changed to topotecan and cranial irradiation was performed. At the same time, a right thyroid nodule was detected on computed tomography of his chest and showed growth in size in the following 4 months. A palpable right thyroid nodule came to our attention at that time, the 10th month of presentation. Free thyroxine, free triiodothyronine, thyroid-stimulating hormone, antithyroglobulin and antithyroid peroxidase antibodies were within normal limits. Thyroid ultrasonography showed a right thyroid lobe 26.2×16.8×15.7mm hypoechoic solid nodule with irregular borders. Ultrasonography-guided thyroid fine-needle aspiration biopsy showed metastasis from small cell lung carcinoma. His cranial metastases worsened. He developed right cervical lymph node, hepatic, pancreatic and meningeal metastases and died 15 months after the initial presentation and 9 months after the detection of thyroid metastasis by computed tomography of his chest. Our case and two previously reported cases were male, 55-years old or older and had history of more than 40 pack-years of cigarette smoking. All had metastatic disease elsewhere, when the thyroid metastasis was diagnosed by fine-needle aspiration biopsy. All had poor survival, between 9 and 18 months, after thyroid metastasis was diagnosed.

**Conclusions:**

We conclude that in a patient with a known history of malignant disease, the finding of a new thyroid mass should be promptly evaluated with a thyroid fine-needle aspiration biopsy to search for metastatic disease. The clinical features of our and two previously reported cases were summarized.

## Introduction

Although the thyroid gland has a rich vascular supply, carcinomas rarely metastasize to the thyroid [1]. The primary carcinomas that metastasize to the thyroid are renal cell (48.1%), colorectal (10.4%), lung (8.3%) and breast (7.8%) carcinomas, melanoma (4%), sarcoma (4%) and other types (17.4%) [2]. Among the lung carcinomas metastasizing to the thyroid, adenocarcinomas are the commonest followed by squamous cell carcinomas [1–5]. Small cell and large cell carcinomas rarely metastasize to the thyroid gland [6–8]. Small cell lung cancer is a rapidly progressive malignancy with a poor survival rate. Small cell lung cancers frequently metastasize to lymph nodes, liver, adrenal glands, bone, brain and pleura. Metastasis of small cell lung cancer to the thyroid is a very rare clinical encounter [6, 7]. We report a case of small cell lung carcinoma that metastasized to the thyroid gland and review the clinical literature.

## Case presentation

**Fig. 1:**
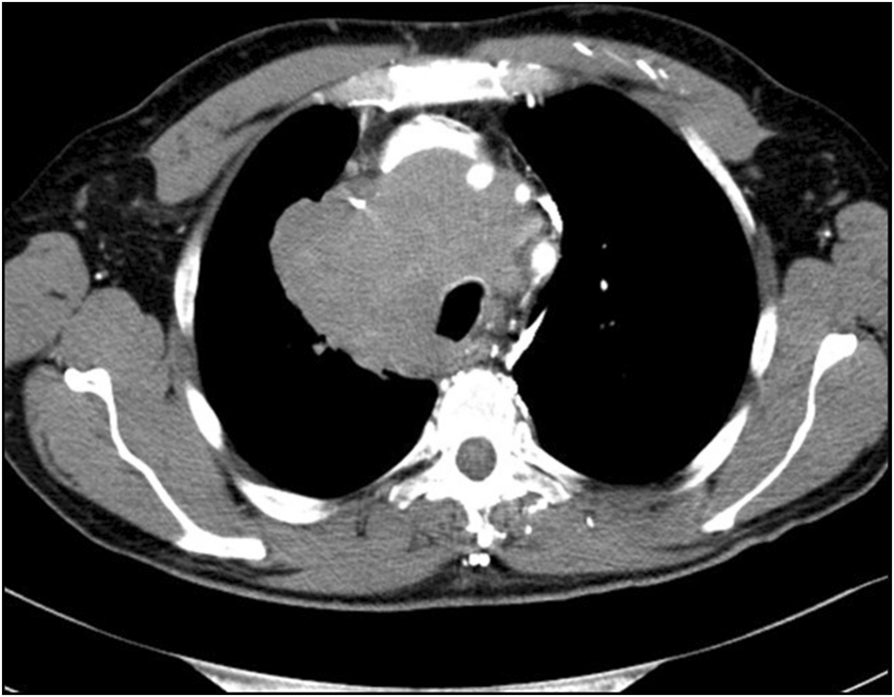
Computed tomography image of the mediastinal mass intermingled with mediastinal lymphadenopathy

**Fig. 2:**
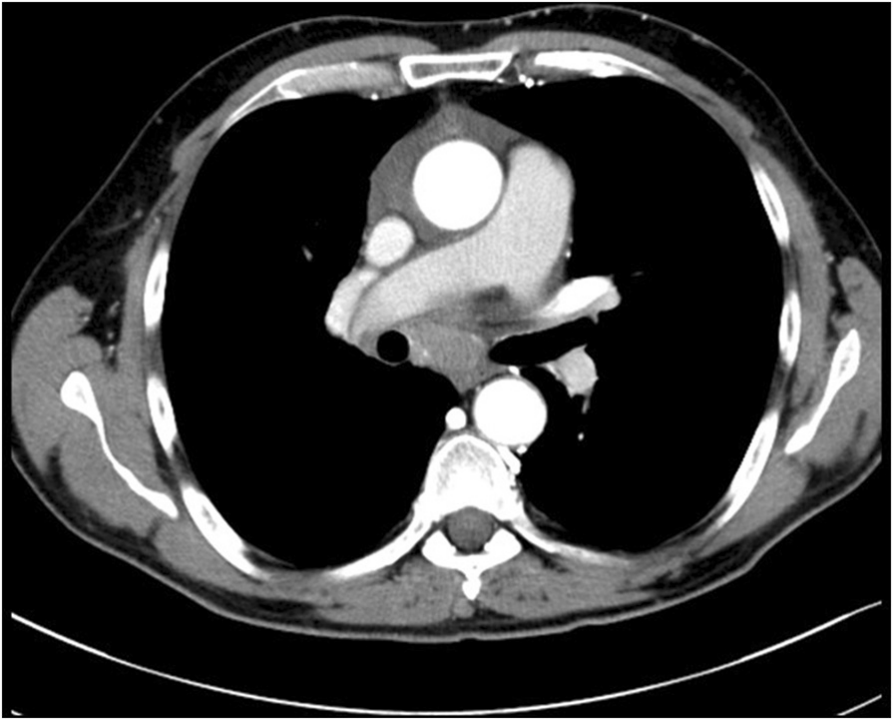
Computed tomography image of subcarinal lymphadenopathy

**Fig. 3:**
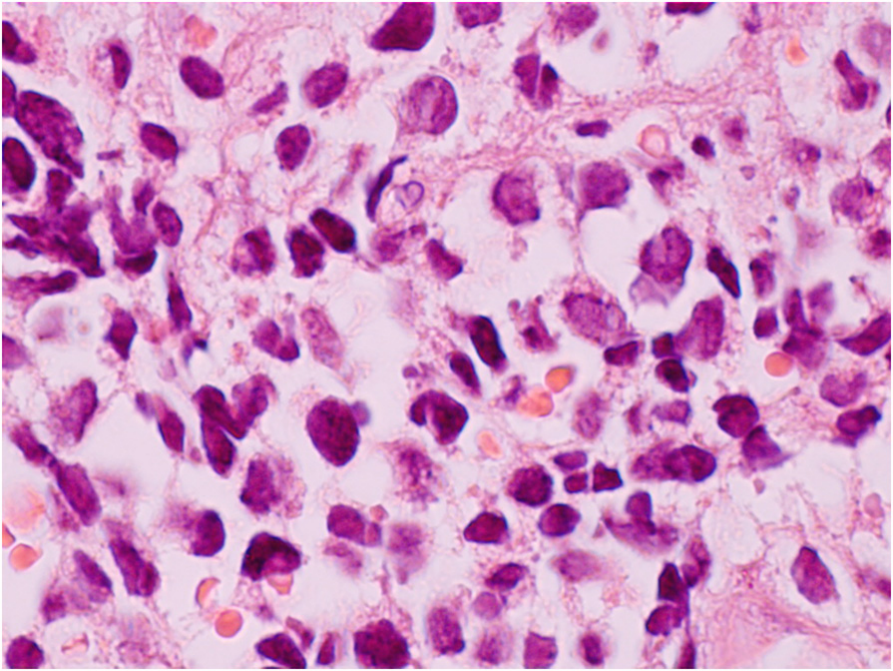
Oil immersion, high power (×1000 magnification) hematoxylin and eosin staining of bronchial biopsy. Small cell carcinoma cells are seen in the lamina propria juxtaposed to red blood cells. Small cell carcinoma cells are characterized by hyperchromatic nuclei, high nuclear to cytoplasmic ratio and nuclear molding

**Fig. 4:**
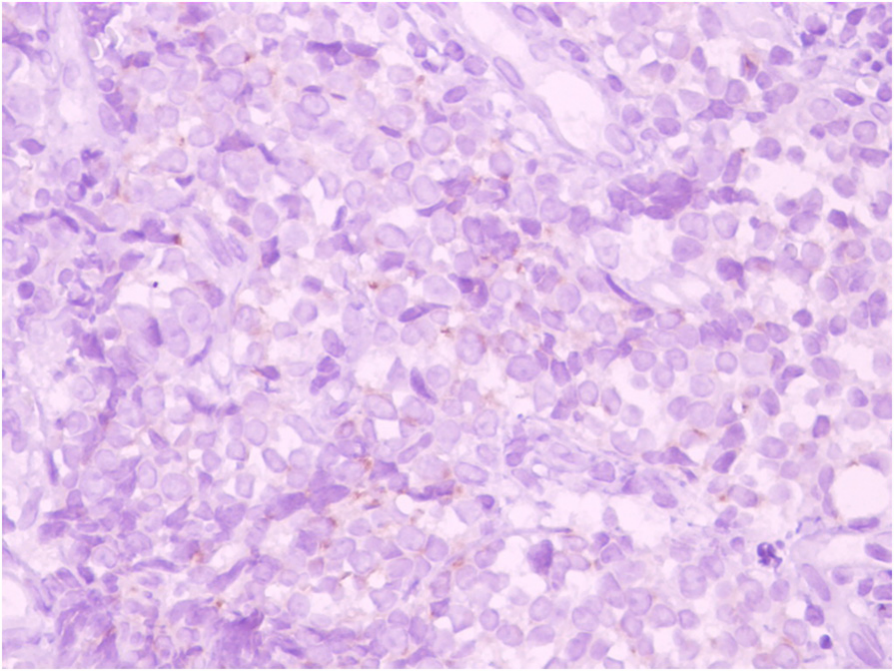
Immunohistochemical (×400 magnification) chromogranin staining (*brown*) as a marker for neuroendocrine differentiation in bronchial biopsy

**Fig. 5:**
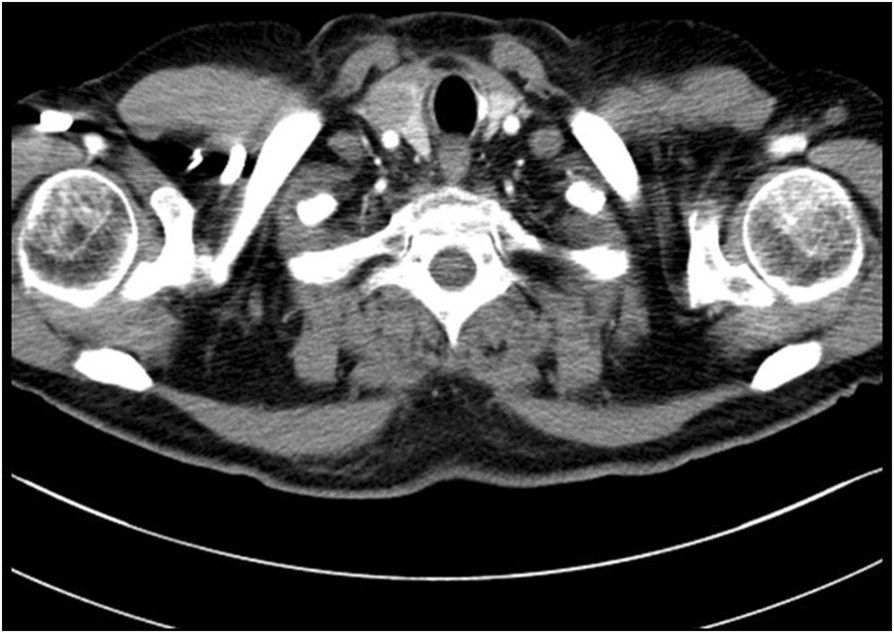
Computed tomography image of the thyroid gland and right thyroid nodule

**Fig. 6:**
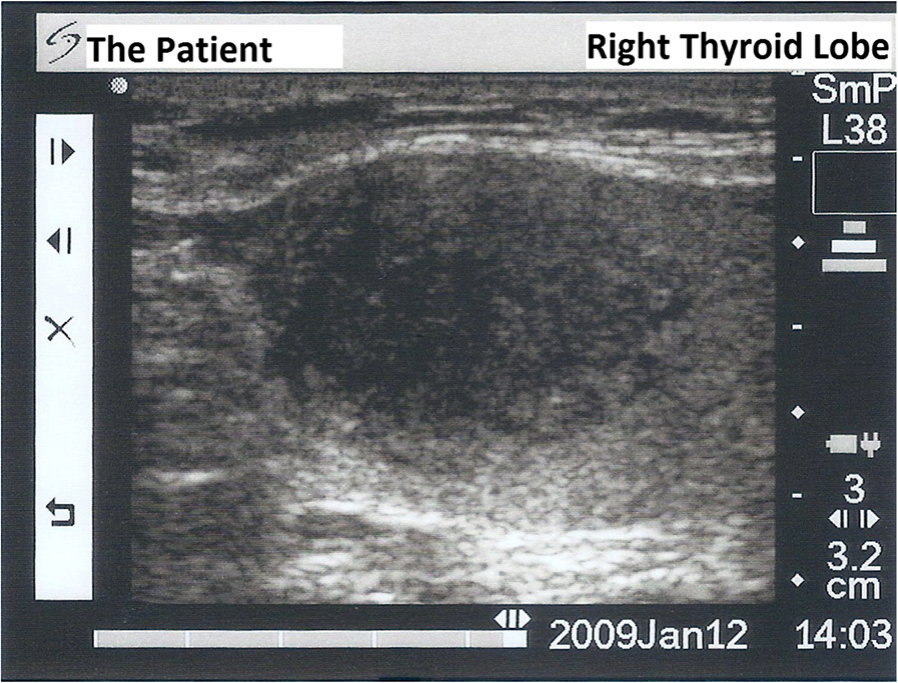
Thyroid ultrasonography image of right thyroid lobe

**Fig. 7:**
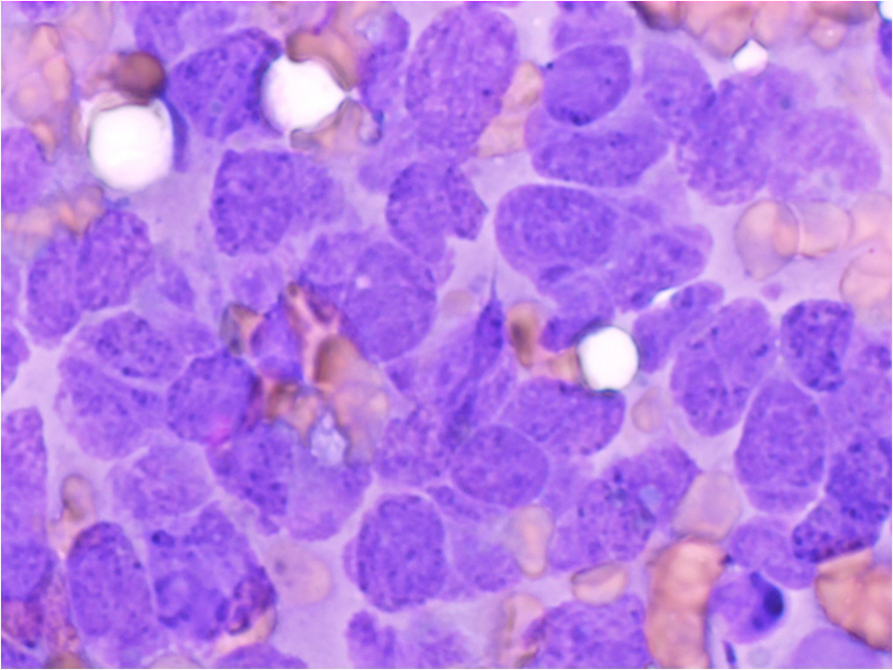
Oil immersion, high power (×1000 magnification) Diff-Quick staining of thyroid fine-needle aspiration biopsy. Small cell carcinoma cells are characterized by hyperchromatic nuclei, coarse chromatin, salt and pepper appearance, high nuclear to cytoplasmic ratio and nuclear membrane irregularities. No thyroid follicular cells are seen. Small cell carcinoma cells are much larger than thyroid follicular cells

A 55-year-old Turkish man presented with swelling in the anterior of his neck and dyspnea for 3 to 4 days. His past medical history was significant for hypertension, hyperlipidemia and a hip prosthesis. He was treated with atenolol, quinapril-hydrochlorothiazide combination and atorvastatin tablets. He had a 50-pack year history of cigarette smoking. His blood pressure was 140/88mmHg, pulse 96 beats per minute and respiratory rate 16 per minute. A chest X-ray showed a mediastinal mass. Computed tomography (CT) of his thorax showed a 10.6×9.7×10.8cm mediastinal mass encasing superior vena cava, causing stenosis of the lumen and deviation of his trachea, esophagus and vascular structures. The mass also reached to the right pulmonary hilus. The presence of lymph node metastasis was not clearly discernible from the images, because the mass appeared somewhat homogeneous (Fig. 1). Subcarinal lymphadenopathy was evident (Fig. 2). In retrospect, we think that the mass was intermingled with multiple lymph node metastases, because they regressed with chemotherapy and radiotherapy. The thyroid appeared normal on CT of his chest. He exhibited clinical features of superior vena cava syndrome. The staging was done by CT of his chest and abdomen. Positron emission tomography-CT and endobronchial ultrasound were not performed. There was no evidence of liver or pancreatic metastases on the initial CT of his abdomen. He had a 15mm hypodense nodule in his left adrenal gland that was not biopsied and remained stable in size during follow-up. A bronchoscopic biopsy showed small cell carcinoma of the lung (Fig. 3). Immunohistochemical staining with synaptophysin, pan cytokeratin and chromogranin (Fig. 4) were positive. A diagnosis of limited stage small cell lung carcinoma was made and he was started on chemotherapy with cisplatin and etoposide. When his tumor diminished in size and fitted to the radiotherapy field, external radiotherapy was instituted. The clinical course showed refractoriness to treatment. He developed radiation pneumonitis and later fibrosis in his right lung. Six months after the presentation, a right thyroid nodule was detected on CT of his chest and multiple brain metastases were detected on magnetic resonance imaging of his brain. Chemotherapy was changed to topotecan. Cranial irradiation was given. He developed pancytopenia. Ten months after the presentation, the patient’s oncologist (second author Gülistan Köksal, MD) was alerted by the finding of the size progression of the right thyroid nodule on a CT scan of the patient’s chest (Fig. 5). On physical examination, a right thyroid nodule was palpable. Thyroid function tests were within normal limits at that time, as well as antithyroglobulin and antithyroid peroxidase antibodies, which were performed at a later date. Tests revealed: free thyroxine (T4) 1.32ng/ml (normal range 0.8 to 1.81), free triiodothyronine (T3) 2.35pg/ml (normal range 2.30 to 4.40), thyroid-stimulating hormone 0.313μIU/ml (normal range 0.27 to 4.20), antithyroid peroxidase antibody 1.1IU/ml (normal range <12), and antithyroglobulin antibody 5.9IU/ml (normal range <34). Thyroid ultrasonography showed a 26.2×16.8×15.7mm hypoechoic solid nodule with irregular borders in his right thyroid lobe (Fig. 6). An ultrasonography-guided thyroid fine-needle aspiration biopsy showed metastasis from small cell lung carcinoma (Fig. 7). His cranial metastases worsened. There was no size reduction of the initially detected metastatic thyroid nodule and two new hypoechoic nodules of 4.2×4.7×2.5mm and 4.2×3.4×3.4mm with blurred borders were detected by thyroid ultrasonography performed 4.5 months after the diagnosis of the thyroid metastasis by fine-needle aspiration biopsy. He developed right cervical lymph node, hepatic, pancreatic and meningeal metastases and expired 15 months after the initial presentation and 9 months after the detection of thyroid metastasis on CT of his chest.

## Discussion

The clinical detection of thyroid metastasis from nonthyroid primary tumors is an extremely rare event, but autopsy series report somewhat higher rates. In a postmortem study performed on patients who died of advanced cancer, the rate of metastasis to the thyroid gland was 8.6% [9]. A meticulous autopsy study (Reference [10] could not be located in PubMed, Scopus, Science Direct and Turkish Academic Network and Information Center and was cross-referred from reference [11]) that microscopically examined at least one slice of thyroid for every 5 gram of tissue in patients who died of metastatic cancer reported the rate of thyroid metastasis as 24.2% [10, 11]. The clinical finding of thyroid metastasis from a nonthyroid primary tumor is much less common, although the incidence depends on how vigorously this is sought. Thyroid metastasis of nonthyroid primary cancers represented 0.16% of all thyroid fine-needle aspiration biopsies and 1.9% of all thyroid fine-needle aspiration biopsies that were categorized as malignant in a multicenter study from the USA and Europe [1]. In an analysis of thyroidectomy specimens performed on patients with a solitary thyroid metastasis (patients with multiple metastases to other sites were excluded), Calzolari *et al*. reported the prevalence of intrathyroid metastasis as 0.15% [12]. Chung *et al*. reviewed secondary thyroid tumors reported between 2000 and 2010, and concluded that among the 293 patients with adequate data, 60% of cases had extrathyroid metastases, either diagnosed previously or concomitantly with thyroid metastasis [2]. Up to 40% of metastases were solitary to the thyroid gland and presented as isolated thyroid metastasis [2]. In only 20% of the cases were the diagnosis of nonthyroid primary cancer and the detection of thyroid metastases concurrent [2]. There are only a few reported cases of thyroid metastasis from small cell lung cancer [6, 7]. Therefore, we decided to report this particular case and review similar cases in the clinical literature. Our literature review focused on articles published in the English language and excluded autopsy series and a case reported earlier in another language [13].

We only found two previous reports of small cell lung cancer metastasis to the thyroid [6, 7]. The clinical features of those and our case are summarized in Table 1. All the patients with thyroid metastasis of small cell lung cancer were male and were 55-years old or older. All had a history of heavy cigarette smoking. All had extensive stage disease when thyroid metastasis was diagnosed. The two previously reported patients had synchronous metastasis to the thyroid gland. Our case had metachronous detection of thyroid metastasis. In patients with metachronous metastases, the mean interval for detection of thyroid metastasis from primary tumors was 68 months for renal cell cancer, 48 months for breast cancer, 42 months for colorectal cancer and 21 months for malignant melanoma [2]. The time period between the diagnosis of primary tumor and the discovery of thyroid metastasis was shortest in patients with lung cancer, with a mean of 4.5 months [2]. In our patient, thyroid metastasis appeared 6 months after the presentation.

**Table 1:** Clinical features of the current and previously reported* cases of small cell lung carcinoma with thyroid metastasis

Author, year, reference	Ozgu et al. 2012 [6]	Katsenos et al. 2013 [7]	Can and Köksal 2015 (this report)
Gender	Male	Male	Male
Age (years)	66	55	55
Comorbidities	CAD, CABG	CAD, PTCA, DM	HTN, HL, hip prosthesis
Smoking history	75 pack-year	40 pack-year	50 pack-year
Thyroid status	Hyperthyroid due to toxic MNG	Euthyroid†	Euthyroid
Other sites of metastasis‡	Adrenal mets	Cervical and mediastinal LN, cerebellar mets	Cerebral mets
Synchronicity	Synchronous	Synchronous	Metachronous
Time to detection of metastasis	NA	NA	6 months
Diagnosis of thyroid metastasis	FNA	FNA	FNA
Thyroidectomy	No	No	No
Treatment	Chemotherapy	Chemotherapy, cranial irradiation	Chemotherapy, lung and cranial irradiation
Survival after diagnosis of thyroid metastasis	11 months	18 months†	9 months

*CABG* coronary artery bypass grafting, *CAD* coronary artery disease, *DM* diabetes mellitus, *FNA* fine-needle aspiration biopsy of the thyroid, *HL* hyperlipidemia, *HTN* hypertension, *LN* lymph nodes, *mets* metastasis, *MNG* multinodular goiter, *NA* nonapplicable because of synchronous diagnosis of the small cell lung cancer and thyroid metastasis, *PTCA* percutaneous transluminal coronary angioplasty, *Literature review was based on clinical cases (not autopsies) and to articles published in the English language †personal e-mail communication with Stamatis Katsenos, MD, PhD on 16 November 2014, ‡represents other sites of metastasis when thyroid metastasis was diagnosed

Our patient was euthyroid without evidence of autoimmune thyroid disease. Ozgu *et al*. reported the coexistence of toxic multinodular goiter and thyroid metastasis from small cell lung cancer in a patient with thyrotoxicosis [6]. Primary nonthyroid tumors may also cause thyrotoxicosis due to massive metastasis causing destructive thyroiditis [14].

In all three reported patients with thyroid metastasis of small cell lung cancer, the diagnosis was established by thyroid fine-needle aspiration biopsy (Table 1). In their recent review of thyroid metastases from nonthyroid malignancies, Chung *et al*. estimated that thyroid fine-needle aspiration biopsy provided the correct diagnosis in 73.7% of patients [2]. Pusztaszeri *et al*. reported that thyroid fine-needle aspiration biopsy categorized 87% of thyroid metastasis of nonthyroid primary carcinomas to malignant or to the suspicious for follicular neoplasm group, the categories that call for surgical intervention as the treatment approach [1]. These sensitivity rates for secondary carcinomas are similar to the sensitivity rate of 83% (range 65 to 98%) for thyroid fine-needle aspiration biopsy for detection of primary carcinomas in thyroid nodules [15]. The difficulty of distinguishing primary thyroid anaplastic carcinoma or poorly differentiated thyroid carcinoma from metastatic high-grade malignancy is reported to be a source of error for accurate cytological interpretation in the evaluation of secondary thyroid tumors [1, 5].

None of the cases of thyroid metastasis from small cell lung carcinoma underwent thyroidectomy (Table 1). All were treated with chemotherapy consisting of cisplatin and etoposide or chemotherapy and radiotherapy. Surgery was not considered in our case, because there were cerebral metastases and surgical excision is not a recommended modality in the management of small cell lung carcinoma. Osawa *et al*. reported the case of a 67-year-old man diagnosed with stage IIIA (T2N2M0) small cell lung cancer who was treated with cisplatin and etoposide followed by cranial irradiation [8]. Four years later, an isolated thyroid metastasis was diagnosed. A thyroid metastasectomy showed large cell carcinoma. Review of the initial lung material showed both small and large cells in the tumor. The authors concluded that the patient had a combined small and large cell carcinoma in his lung as the primary, and the large cell component metastasized to the thyroid gland [8]. As the large cell carcinoma component metastasized to the thyroid, we did not include this case in Table 1.

The appearance of thyroid metastasis is thought to indicate a poor prognosis. Earlier case reports or series presented the average survival from diagnosis to death as 2 months in lung carcinomas with thyroid metastasis [4, 11]. The survival of the recently reported patients in 2012 and 2013 and our patient ranged between 9 and 18 months (Table 1 and personal e-mail communication with Stamatis Katsenos, MD, PhD on 16 November 2014) and probably reflects the effect of improved treatment modalities in the last few years compared to previous decades. From a case series of 25 patients with solitary intrathyroid metastasis, Calzolari *et al*. reported that patients with a single metastatic lesion on the thyroid from a nonthyroid primary carcinoma had better survival than those with multiple metastases to the thyroid [12].

The incidence of primary thyroid cancer is on the rise [16], but it is not known if the incidence of thyroid metastases from nonthyroid malignancies is also on the rise. There is an increase in the number of case reports of nonthyroid primary cancer metastasis to the thyroid gland. From the Mayo Clinic, only two cases of thyroid metastasis from nonthyroid primary cancers were observed in thyroid surgeries performed between 1892 and 1932 [17], 14 cases were reported from the preceding 55-year period in 1964 [17], 12 cases during the preceding 21-year period in 1982 [18], 15 cases during the preceding 6-year period in 1987 [19], and 39 cases in the preceding 10-year period in 1997 [5]. The upper limit of the preceding years ended a few years earlier than the publication dates of the Mayo Clinic reports. In 2010, Chung *et al*. reported 372 cases of thyroid metastases of nonthyroid malignancies and pointed out that there is an apparent increase in the number of reported cases of thyroid metastasis from nonthyroid primary tumors [2]. This observed increase can be attributed to the growth of the population [20] resulting in an increasing number of cases of cancer and thus metastasis, an increase in the number of journals facilitating publication [21] or an increase in the incidence of nonthyroid cancer [22]. Increasing use of imaging technologies, such as positron emission tomography, more frequent use of thyroid fine-needle aspiration biopsy and improved survival of patients with disseminated cancer probably contributed to the increased number of case reports of thyroid metastasis from nonthyroid primary carcinomas [1, 2].

Primary small cell carcinomas originating from the urinary bladder [23] and uterine cervix [24] have also been reported to metastasize to the thyroid gland. Primary small cell carcinoma of the thyroid is a very rare entity [25]. After the introduction of immunohistochemistry, some of the previously diagnosed primary small cell carcinomas of the thyroid were diagnosed as primary lymphomas, poorly differentiated insular carcinomas of the thyroid or small cell variants of medullary carcinomas [25]. A variety of neuroendocrine tumors have also been reported to metastasize to the thyroid [26].

In view of our clinical experience of this reported case, we suggest that the finding of a new thyroid nodule in a patient with a known malignancy should trigger a search for metastasis. Secondary thyroid tumors after a nonthyroid primary tumor are rare. Ultrasonography-guided thyroid fine-needle aspiration biopsy aids in the diagnosis and helps to differentiate whether the nodule in question is a benign thyroid nodule, a primary thyroid carcinoma or metastatic thyroid carcinoma.

## Conclusions

We report a rare case of thyroid metastasis from small cell lung carcinoma. After reviewing previously reported cases [6, 7] and our case, we conclude that the appearance of a thyroid nodule during follow-up of patients with a prior history of malignancy should be promptly evaluated with a thyroid fine-needle aspiration biopsy to search for metastatic disease.

## Consent

Written informed consent was obtained from the patient for publication of a case report and accompanying images. A copy of the written consent is available for review by the Editor-in-Chief of this journal.
